# Recalibration of vocal affect by a dynamic face

**DOI:** 10.1007/s00221-018-5270-y

**Published:** 2018-04-25

**Authors:** Martijn Baart, Jean Vroomen

**Affiliations:** 1 Department of Cognitive Neuropsychology, Tilburg University, P.O. Box 90153, 5000 LE Tilburg, The Netherlands; 2 BCBL, Basque Center on Cognition, Brain and Language, Donostia, Spain

**Keywords:** Emotion perception, Cross-modal learning, Audiovisual integration, Adaptation

## Abstract

Perception of vocal affect is influenced by the concurrent sight of an emotional face. We demonstrate that the sight of an emotional face also can induce recalibration of vocal affect. Participants were exposed to videos of a ‘happy’ or ‘fearful’ face in combination with a slightly incongruous sentence with ambiguous prosody. After this exposure, ambiguous test sentences were rated as more ‘happy’ when the exposure phase contained ‘happy’ instead of ‘fearful’ faces. This auditory shift likely reflects recalibration that is induced by error minimization of the inter-sensory discrepancy. In line with this view, when the prosody of the exposure sentence was non-ambiguous and congruent with the face (without audiovisual discrepancy), aftereffects went in the opposite direction, likely reflecting adaptation. Our results demonstrate, for the first time, that perception of vocal affect is flexible and can be recalibrated by slightly discrepant visual information.

## Introduction

Successful human interaction hinges on our ability to perceive emotions, which are prevalent in body movements (Darwin 1872), faces (e.g., Ekman et al. 1987), and voices (e.g., Scherer et al. 2001). Recognition of basic vocal (e.g., Beier and Zautra 1972) and non-verbal (e.g., Sauter et al. 2010) emotions is robust across cultures, and emotional information is often multisensory. This multisensory nature of emotional affect can lead to cross-modal capture effects where information in one modality can affect the perceived emotion in another modality (e.g., de Gelder and Vroomen 2000; Dolan et al. 2001). For example, a ‘fearful’ voice is more likely to be perceived as ‘fearful’ if accompanied by a ‘fearful’ face rather than a ‘happy’ one, and emotionally congruent audiovisual stimuli are responded to faster than incongruent ones (e.g., Dolan et al. 2001). This type of integration happens automatically (e.g., Föcker et al. 2011; Vroomen et al. 2001) with quick neural consequences (e.g., Pourtois et al. 2000).

Prolonged exposure to small intermodal conflicts can also induce assimilative aftereffects. For example, exposure to discordant visual and proprioceptive information (when the perceived location of a hand is displaced when looking through a prism) results in aftereffects in both visual and proprioceptive localization (Welch 1986). Similar aftereffects are observed with audiovisual discordant spatial information (Radeau and Bertelson 1974), temporal information (Fujisaki et al. 2004; Vroomen et al. 2004), and phonetic information (e.g., Baart and Vroomen 2010; Bertelson et al. 2003; Vroomen and Baart 2012). These aftereffects show that exposure to conflicting inputs can recalibrate processing in the respective modalities, such that the conflict between the modalities is reduced. It is generally agreed that recalibration helps to maintain coordinated operations in an environment where sensory inputs are subject to change because of spontaneous drift, growth, or sensory handicaps (de Gelder and Bertelson 2003).

**Fig. 1:**
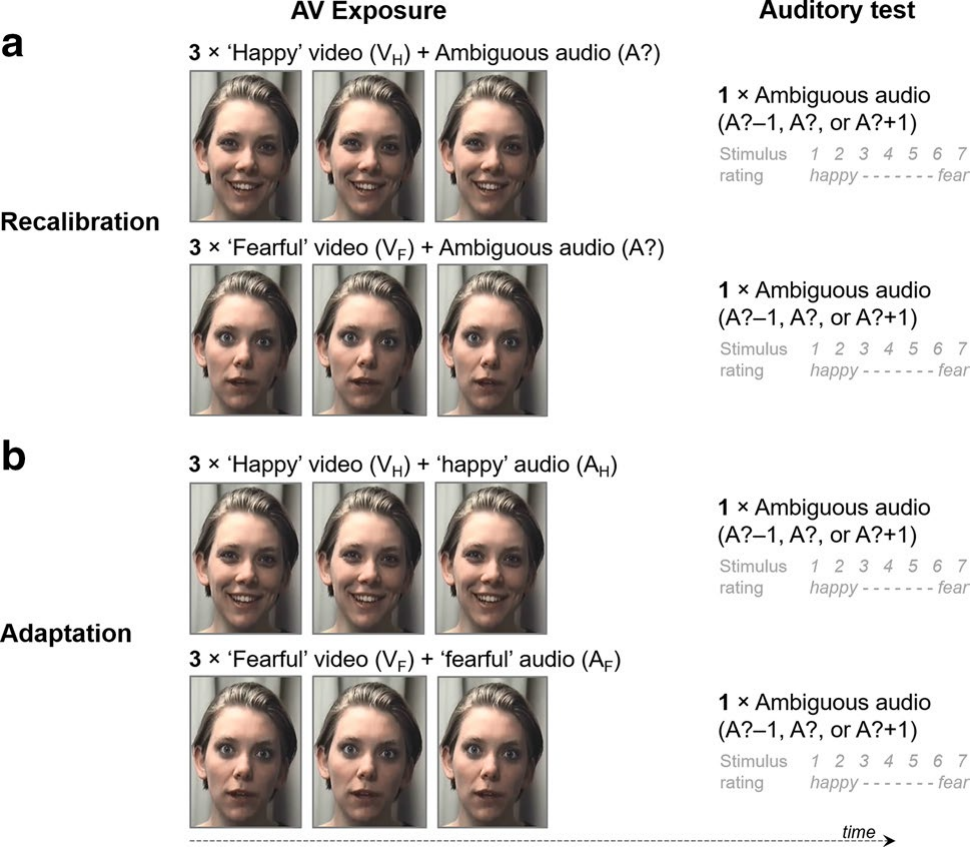
Overview of the audiovisual exposure—auditory test design. Recalibration (**a**): three repetitions of a dynamic video of a ‘happy’ or ‘fearful’ speaker pronouncing an auditory sentence with ambiguous emotional auditory prosody were followed by an auditory-only test in which one out of three ambiguous sentence was rated for emotional affect. Exposure stimuli with ambiguous prosody were expected to induce assimilative aftereffects (recalibration) because the video shifts the interpretation of the ambiguous sound so that the audiovisual conflict is reduced. Adaptation (**b**): the procedure was the same as in **a**, except that the exposure stimuli had auditory sentences with non-ambiguous happy or fearful prosody that were congruent with the video. These stimuli were expected to induce contrastive after effects because the non-ambiguous nature of the sentences induces adaptation

Although cross-modal biases in emotion perception are well-known, aftereffects indicative of cross-modal recalibration of affect have never been demonstrated, despite the fact that their relevance for social interaction seems obvious. Here, we used dynamic audiovisual stimuli to determine whether recalibration occurs in the domain of emotion perception. We exposed listeners to an auditory sentence whose prosody was halfway between a ‘happy’ and ‘fearful’ emotion^1^ (A?, for auditory ambiguous) in combination with a dynamic and synchronized video of a speaker pronouncing this sentence in either a ‘happy’ or ‘fearful’ way (V_H_ and V_F_, for visual ‘happy’ and ‘fearful’, respectively). Following exposure to these audiovisual sentences, listeners rated the valence of auditory-only sentences with (somewhat) ambiguous prosody (i.e., test items were A?, the more ‘fearful-like’ A? − 1 item, and the more ‘happy-like’ A + 1 item, see Fig. 1a). We expected that the video of the face would recalibrate the perceived emotion of the auditory sentence so that the rating of subsequent auditory-only test trials would be shifted towards the emotional state of the previously seen video. Participants would thus rate an auditory ambiguous sentence as more ‘happy’ if during the previous exposure phase it was combined with a ‘happy’ face rather than a ‘fearful’ face.

To rule out the possibility that this shift is a carry-over effect (or priming) of seeing a ‘happy’ or ‘fearful’ expression during exposure, we included a control condition, as in Bertelson et al. (2003, Experiment 2), in which the auditory exposure sentence was emotionally non*-*ambiguous and congruent with the face (see Fig. 1b). Despite the fact that the visual information in these non-ambiguous exposure stimuli is identical to the ambiguous ones, we did not expect assimilative aftereffects (or recalibration), because there is no audiovisual conflict that needs to be resolved. Instead, the audiovisual congruent face/voice pairings were expected to produce contrastive aftereffects, or adaptation, and the ambiguous test sentences were thus expected to be perceived less in accordance with the visual emotion seen during exposure. This effect may be driven by the non-ambiguous auditory information (e.g., Diehl et al. 1980), or because there is supra-modal adaptation in which case exposure to facial affect itself may elicit (relatively small) aftereffects in the perception of vocal affect (e.g., Skuk and Schweinberger 2013).

## Materials and methods

### Participants

27 Tilburg University students participated in return for course credits. All participants had (corrected to) normal vision, adequate hearing and no known neurological disorders. Written informed consent was obtained prior to testing. The study was conducted in accordance with the Declaration of Helsinki, and approved by the Tilburg University ethical committee (project ID: EC-2016.48). Three participants were excluded from analyses (see Results, Valence ratings of the auditory 7-step continuum). Mean age of the remaining 24 participants (17 females) was 19.29 (SD = 1.73).

### Stimuli

**Fig. 2:**
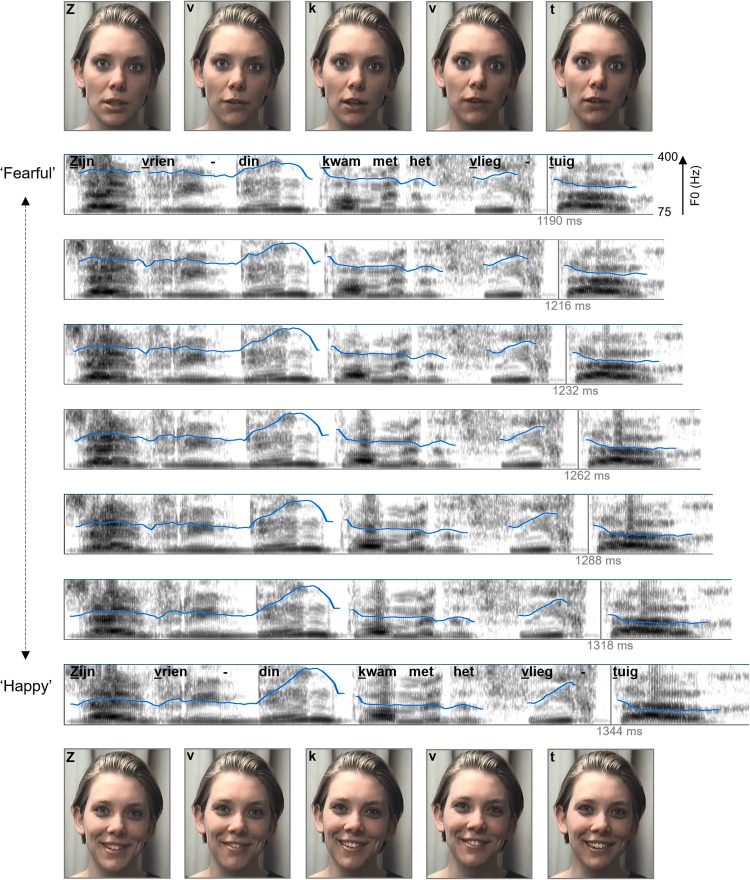
Stimulus overview. The pitch contour of the seven sentences are indicated by the blue line (on a 75–400 Hz scale), and are superimposed on the spectrograms (0–5000 Hz, 50 dB dynamic range). Relative timing of the auditory sentence is indicated by the text in the spectrograms of the ‘fearful’ and ‘happy’ continuum endpoints. The underlined letters correspond to the video frames that are provided above/below the spectrograms

The auditory stimuli comprised seven tokens on a ‘happy’-to-‘fearful’ auditory continuum of the semantically neutral Dutch sentence “Zijn vriendin kwam met het vliegtuig” (His girlfriend arrived by plane). The stimuli are described in detail in de Gelder and Vroomen (2000). In short, the ‘happy’ sentence served as the ‘source-signal’ whose average fundamental frequency (corresponding to the perceived pitch level), excursion size of the fundamental frequency of the accented words (‘vrienDIN’ and ‘VLIEGtuig’), and duration (which are critical prosodic parameters that convey affect, see Vroomen et al. 1993) were shifted towards typical ‘fearful’ parameters in 6 steps with PSOLA (‘pitch synchronous overlap and add’ method). This resulted in a high-quality 7-step prosodic continuum from ‘happy’ to ‘fearful’ (see Fig. 2). Pre-tests showed that the fourth token of the continuum had the most ambiguous emotional valence (denoted as A?, for the auditory most ambiguous sentence).

The video recordings were made by recording another speaker (i.e., all sentences were dubbed) who pronounced the same sentence while displaying a ‘happy’ or a ‘fearful’ state, (i.e., smiling and relaxed expression vs. wide-eyed and worried expression). Four audiovisual exposure stimuli were then created: Two consisted of A? dubbed onto the ‘happy’ and ‘fearful’ videos (A?V_H_ and A?V_F_), and two were emotionally congruent AV pairings (A_H_V_H_ and A_F_V_F_). The audiovisual exposure stimuli looked and sounded natural without any noticeable delay, and can be retrieved from: http://www.martijnbaart.com/avemotion.zip.

### Design and procedure

The study comprised three subsequent phases: (1) rating of the valence of the auditory stimuli of the 7-step continuum, (2) rating of the auditory valence of the three most ambiguous auditory sentences when preceded by audiovisual exposure stimuli meant to induce recalibration or adaptation, and (3) rating of the auditory valence of each of the four audiovisual exposure stimuli.

The experiment was run in a dimly-lit and sound-attenuated booth. Stimuli were delivered using the E-prime 3.0 software. Audio was presented at ~ 65 dB via two speakers (Altec Lansing, ADA215) placed underneath the monitor (BenQ Zowie XL 2540), which was set at a resolution of 1920 × 480 px (full HD) at a refresh-rate of 240 Hz. Participants were seated at ~ 60 cm from the monitor, and size of the videos was 16.5 cm (W) × 13.5 (H) cm. Total testing lasted ~ 30 min.

*Valence ratings of the auditory 7-step continuum* Participants were first acquainted with the two endpoints of the continuum (each of the extremes was presented twice). Next, they rated the emotional valence of each continuum sentence on a 7-point scale from 1 (‘happy’) to 7 (‘fearful’), by pressing the corresponding key on a keyboard. Each of the seven sentences was presented 8 times in random order for a total of 56 trials. Each trial started with a fixation cross that remained on the screen during the sentence. Responses were collected after the sentence ended.

*Valence ratings of the middle 3 auditory test stimuli after audiovisual exposure* In total, there were 96 exposure–test mini-blocks presented in random order, divided over two sessions of 48 mini-blocks with a self-paced break in between. Half of the mini-blocks contained audiovisual exposure stimuli with ambiguous prosody (A?V_H_ and A?V_F_), the other half contained audiovisual stimuli with non-ambiguous and congruent prosody (A_H_V_H_ and A_F_V_F_).

During an exposure–test block, participants saw a 500 ms fixation cross, which was followed by three repetitions of one of the 4 audiovisual exposure stimuli. An auditory test sentence followed 750 ms after the end of the last exposure video. The test sentence was either A?, the more ‘happy-like’ A?+1 sentence, or the more ‘fearful-like’ A?-1 sentence of the continuum. Participants rated the valence of the test sentence using the same procedure and response scale as before. There were 24 blocks for each exposure stimulus, 8 for each of the three test sentences.

*Valence ratings of the audiovisual exposure stimuli* Each exposure stimulus was presented 8 times (in random order), for a total of 32 trials, all preceded by a fixation cross (500 ms). Participants rated the valence of the auditory part of the audiovisual stimulus on the same 7-point Likert scale. Participants were asked to look at the video, but to base their response on the audio.

## Results

### Valence ratings of the auditory 7-step continuum

**Fig. 3:**
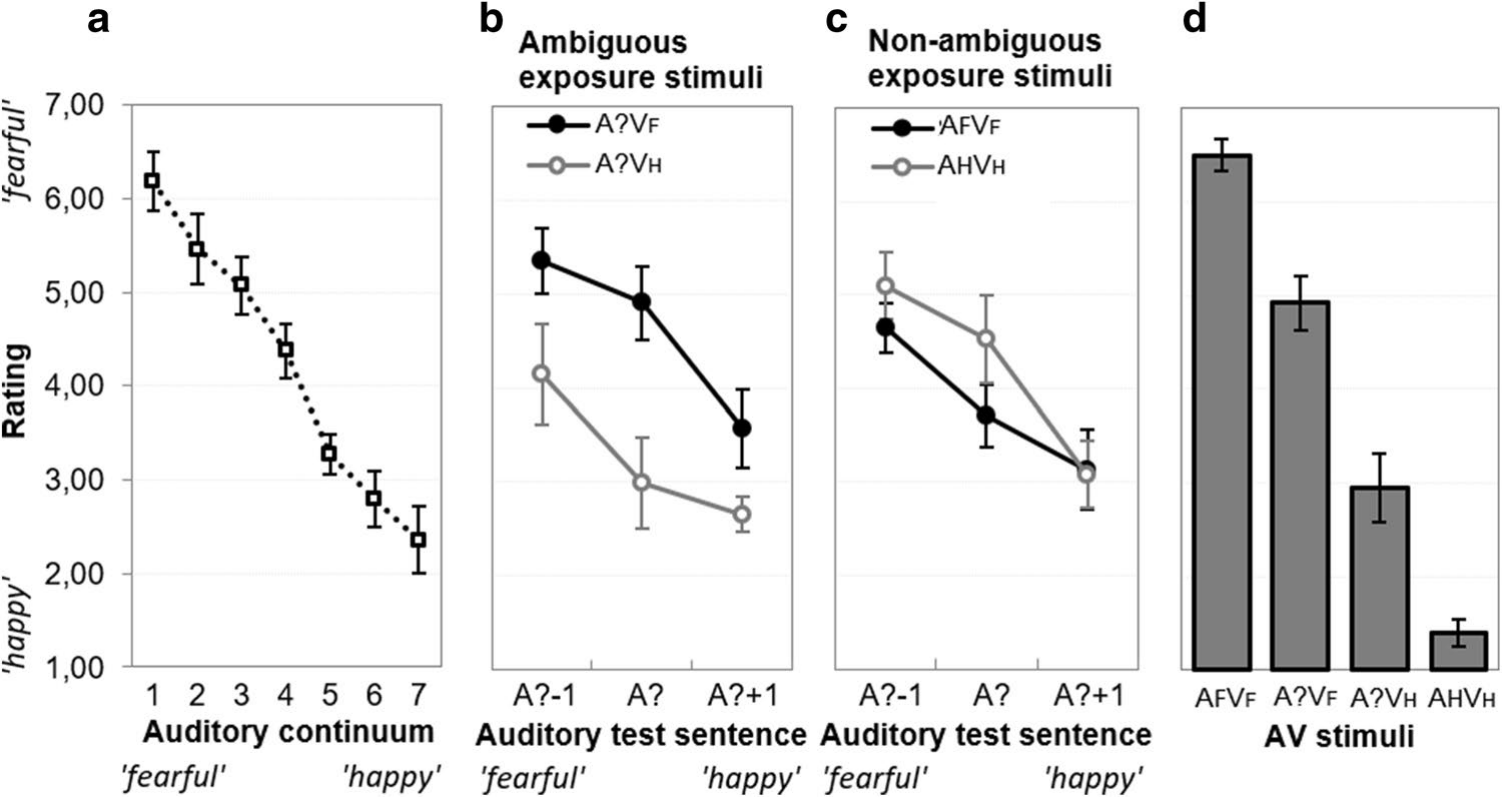
Group-averaged valence ratings of the voice of **a** the auditory-only 7-step continuum, **b** the audio-only test tokens after exposure to audiovisual stimuli with ambiguous and slightly incongruent prosody, **c** the audio-only test tokens after exposure to audiovisual stimuli with non-ambiguous and congruent prosody, **d** the audiovisual exposure stimuli. Error bars represent 95% Confidence Intervals of the mean

Three participants did not perceive the emotional valence in the sentences as intended (their rating difference between the ‘fearful’ and ‘happy’ continuum endpoints was < 1, whereas the mean difference was 3.82 for the other participants), and were excluded from further analyses.^2^ For the remaining 24 participants, Fig. 3a displays the group-averaged ratings of each auditory sentence. The average rating dropped from 6.18 for the ‘fearful’-end of the continuum to 2.36 for the ‘happy’-end of the continuum. A repeated measures ANOVA on the rating of the tokens confirmed that the acoustic manipulations were effective *F*(6,138) = 115.40, *p* < 0.001, *η*^*2*^_*p*_ = 0.834. Follow-up pair-wise *t* tests showed that the differences between the averaged rating scores for adjacent tokens were all significant, *t*s(23) > 2.42, *p*s < 0.024, *d*s > 0.475.

### Valence ratings of the middle 3 auditory test stimuli after audiovisual exposure

A 2 (exposure type: audiovisual incongruent vs. congruent) × 2 (emotion of the video: fear vs. happy) × 3 (prosody of test sentence: A? − 1, A?, A? + 1) repeated measures ANOVA on the valence ratings showed no main effect of exposure type, *F*(1,23) = 1.80, *p* = .193, *η*^*2*^_*p*_ = .073. There was a main effect of emotion of the video, *F*(1,23) = 4.48, *p* = 0.045, *η*^*2*^_*p*_ = 0.163, because mean valence ratings were higher for ‘fearful’ videos than ‘happy’ videos (4.22 vs. 3.75, respectively). There was a main effect of prosody of the test sentence, *F*(2,46) = 92.80, *p* < 0.001, *η*^*2*^_*p*_ = 0.801, because ratings dropped as the test sentences moved from ‘fearful’ towards the ‘happy’ end of the continuum. Critically, there was an interaction between exposure type and emotion of the video, *F*(1,23) = 85.00, *p* < 0.001, *η*^*2*^_*p*_ = 0.787, indicating that the valence ratings of the auditory test sentences were modulated by the preceding exposure phase (see Fig. 3b, c). That is, when the prosody of the exposure stimuli was ambiguous and slightly incongruent, aftereffects were assimilative (i.e., recalibration) and the test sentences were rated more in accordance with the visual emotion seen during exposure (an overall V_F_–V_H_ difference of + 1.35 units, pooled over the three test sentences). In contrast, when the prosody of the exposure stimuli was non*-*ambiguous and congruent, aftereffects were contrastive (i.e., adaptation) and the test sentences were rated less in accordance with the emotion displayed during exposure (an overall V_F_–V_H_ difference of − 0.40 units, pooled over the three test sentences).

There was also an interaction between exposure type, emotion of the video, and prosody of the test sentence, *F*(2,46) = 10.27, *p* < 0.001, *η*^*2*^_*p*_ = 0.309, because these aftereffects were largest for the most ambiguous test sentence A?.

### Valence ratings of the audiovisual exposure stimuli

A 2 (prosody: ambiguous vs. non-ambiguous) × 2 (emotion of the video: fear vs. happy) repeated measures ANOVA on the valence ratings showed a main effect of emotion of the video, *F*(1,23) = 420.47, *p* < 0.001, *η*^*2*^_*p*_ = 0.948, because ratings were higher (more fearful) for stimuli with the ‘fearful’ video (A?V_F_ and A_F_V_F_, mean = 5.71) than for stimuli with the ‘happy’ video (A?V_H_ and A_H_V_H_, mean = 2.17). There was no overall effect of prosody (*F* < 1), but there was an interaction between prosody and emotion of the video, *F*(1,23) = 115.05, *p* < 0.001, *η*^*2*^_*p*_ = 0.833, because the ratings were more extreme for non-ambiguous stimuli. The ratings for stimuli with ambiguous prosody (4.92 vs. 2.94 for A?V_F_ vs. A?V_H_) was significant, *t*(23) = 6.71, *p* < 0.001, *d* = 2.55, indicating that the video had ‘captured’ perceived valence of the sound. This is underscored by the difference between the ratings of the ambiguous exposure stimuli, and the same ambiguous sentence presented in isolation: in isolation, mean rating of A? was 4.37, which was *lower* than the mean rating of A?V_F_, *t*(23) = 2.79, *p* = 0.010, *d* = 0.806, and *higher* than the mean rating of A?V_H_, *t*(23) = 6.99, *p* < 0.001, *d* = 1.82. In addition, the video had also affected rating of the auditory extremes: when the ‘fearful’ continuum endpoint (as tested in isolation) was combined with the ‘fearful’ video, ratings became more ‘fearful’ (a 0.32 difference), *t*(23) = 2.26, *p* = 0.034, *d* = 0.508, and when the ‘happy’ continuum endpoint was combined with the ‘happy’ video, ratings became more ‘happy’ (a 0.96 difference), *t*(23) = 5.18, *p* < 0.001, *d* = 1.142.

## Discussion

The principal and novel finding is that we observed cross-modal assimilative aftereffects when an emotional face was combined with a voice that had ambiguous prosody halfway between ‘happy’ and ‘fearful’. Participants rated the valence of a voice with ambiguous prosody as more ‘fearful’ if during a previous exposure phase this sentence was combined with the video of a ‘fearful’ face instead of a ‘happy’ face. Our interpretation of this finding is that during exposure, the video not only ‘captured’ the valence of the voice, but also induced an enduring shift in the interpretation of the voice that reduced the cross-modal conflict. This enduring shift was then observable as an aftereffect on subsequent auditory test trials. This kind of audiovisual recalibration had already been demonstrated for the perception of space, time, and phonetic speech (e.g., Bertelson et al. 2003; Fujisaki et al. 2004; Radeau and Bertelson 1974; Vroomen et al. 2004), but this is the first time that it has been found for vocal affect.

This assimilative aftereffect could not be attributed to a simple carry-over effect of seeing an emotion in the face during exposure, because for audiovisual congruent exposure stimuli—in which the facial information was exactly the same—aftereffects were contrastive, and thus went in the opposite direction. With congruent exposure stimuli, auditory test stimuli were thus rated in accordance with the emotion not seen during exposure. Similar adaptation effects have been reported before by, for example, Skuk and Schweinberger (2013), who showed that emotional judgments of auditory pseudo-words shifted towards ‘happy’ if they were preceded by audiovisual ‘angry’ stimuli, and vice versa. These contrast effects for emotion have also been observed with auditory-only and visual-only stimuli (Bestelmeyer et al. 2010a, b), and are well-known in phonetic perception under the term ‘selective speech adaptation’ (e.g., Eimas and Corbit 1973; Samuel 1986).

It is of interest to note that assimilative and contrastive aftereffects also have been reported in facial identity priming experiments that might bear resemblance to the present results. For example, in a prime (S1)—target (S2) paradigm, Walther, Schweinberger, Kaiser, and Kovács (2013) demonstrated that when presentation of an unambiguous face (S1) was followed by a slightly ambiguous morphed S2 target that closely resembled S1, identity perception of the morphed S2 face was pulled towards S1 (assimilation). In contrast, if the distance between S1 and S2 was larger and facial identity of S2 was more ambiguous, aftereffects became contrastive. ERP data also showed that the time-course of these two effects were different (Walther et al. 2013). This aligns with earlier work that linked the two phenomena (Huber 2008), which is relevant because attempts are also made to explain recalibration and adaptation within a single underlying model (Kleischmidt and Jaeger 2011).

However, unlike these unimodal aftereffects observed by Walther et al. (2013) that critically depend on the S1–S2 distance, we would argue that the assimilative aftereffects we observed here are driven by the bimodal discrepancy in the exposure stimulus. That is, the visual signal in the AV exposure stimuli captures the perceived auditory affect (see Fig. 3d), and this type of capture is exactly what is assumed to lie at the foundation of recalibration: Presumably, the repeated inter-sensory discrepancy during AV exposure is reduced by shifting the auditory interpretation towards the video, and this results in longer-term assimilative shifts that become apparent as assimilative aftereffects. Given the analogy between this effect and, for example, visually driven learning effects that become apparent as adjustments in proprioception after looking through a prism (Welch 1986), we believe the term ‘recalibration’ provides the best explanation for this phenomenon. Nevertheless, we acknowledge that further research is needed to disentangle the contribution of ‘repetition priming’ and ‘cross-modal error reduction’ to the assimilative aftereffects.

Unlike recalibration, adaptation is often argued to result from exposure to unambiguous information in one particular modality that produces contrastive perceptual effects in the same modality (e.g., Roberts and Summerfield 1981). However, adaptation in emotion cannot solely be explained by the acoustic properties of the adapter sound. For example, Bestelmeyer, Rouger et al. (2010) showed that adaptation to acoustically exaggerated caricatured vocal expressions was equal to adaptation observed with normal vocal affect. So despite that the caricatures were acoustically ‘more extreme’ (and were also rated as such), they did not induce stronger adaptation. Furthermore, adaptation for emotional valence can also cross modalities (e.g., Pye and Bestelmeyer 2015), such as when adaptation to a silent ‘happy’ video induces more ‘angry’ responses for auditory test stimuli (Skuk and Schweinberger 2013). Follow-up work is needed to determine whether our adaptation effects were driven by the auditory and/or visual information.

Another interesting direction for future work is to determine the generality of emotional recalibration and its neural correlates. It is known that phonetic recalibration is sub-served by a brain network that includes the superior temporal sulcus (STS, see Bonte et al. 2017; Kilian-Hutten et al. 2011), which is also involved in audiovisual integration of emotional affect (e.g., Ethofer et al. 2006; Hagan et al. 2009; Klasen et al. 2011). Given that comprehension of vocal affect is driven by bilateral mechanisms that involve a myriad of sensory, cognitive, and emotional processing systems (Schirmer and Kotz 2006), it may well be that STS also has a functional role in recalibration of emotional valence. Another neural structure of potential interest is the amygdala. It is involved in unimodal perception of (negative) emotions (e.g., Scott et al. 2010, 1997), audiovisual emotion perception (Dolan et al. 2001; Klasen et al. 2011), and its activity is modulated by emotional presence rather than congruence. That is, activity for incongruent stimuli (such as when a ‘fearful’ face is combined with sounds of laughter) is comparable to congruent stimuli, but when both unimodal signals contain emotion, activity in the left amygdala is stronger than for stimuli where one signal is emotionally neutral (Müller et al. 2011, but see Dolan et al. 2001, where activity in the left (basolateral) amygdala is modulated by emotional congruence). This is particularly interesting because here, we showed that the perceived emotional valence of neutral sounds can be changed through audiovisual exposure. If the emotionally neutral status of a stimulus can be changed via recalibration, it is thus conceivable that the amygdala is involved in this process. Perhaps a less self-evident brain structure of potential interest is the Putamen. Not only is it involved in processing of negative affect such as recall-generated ‘sadness’ (Reiman et al. 1997), and recognizing (and experiencing) ‘disgust’ (Calder et al. 2000), but regional blood flow in the left Putamen is also significantly correlated with the magnitude of participants’ smiling behavior (quantified with electromyography, or EMG) in reaction to a silent funny movie (Iwase et al. 2002). The Putamen is thus involved in processing of negative as well as positive affect, and it may, therefore, prove to be important in general (i.e., not emotion specific) recalibration of emotional affect.

To conclude, we found that a face can induce an enduring assimilative shift in the perception of vocal affect. This recalibration effect was observed if there was a small discrepancy between the affect displayed in the face and voice. When the face and voice were congruent, contrastive aftereffects were found reflecting adaption. This first report paves the way for further research into the neural mechanism of recalibration of vocal affect.

## Footnotes

1 There are many prosodic cues that may convey affect, such as phonation type (e.g., Birkholz et al. 2015), voice quality, loudness, rhythm, pitch, and duration. As described in the Materials and methods section, we manipulated the average fundamental frequency in the pitch register, the excursion size of the fundamental frequency, and overall duration.

2 Including these outliers did not affect any of the patterns of (non)significance reported in the manuscript.
